# Effects of the “Active Communication Education” Program on Hearing-Related Quality of Life in a Group of Italian Older Adults Cochlear Implant Users

**DOI:** 10.3389/fpsyg.2022.827684

**Published:** 2022-05-20

**Authors:** Ilaria Giallini, Maria Nicastri, Bianca M. S. Inguscio, Ginevra Portanova, Giuseppe Magliulo, Antonio Greco, Patrizia Mancini

**Affiliations:** Department of Sense Organs, University Sapienza of Rome, Rome, Italy

**Keywords:** cochlear implant, older adults, rehabilitation, quality of life, hearing loss, communication programs

## Abstract

**Introduction:**

The present study aimed to evaluate the effects of the Active Communication Education (ACE) program on the social/emotional impacts of hearing loss (HL) in a group of older adults with a cochlear implant (CI).

**Design:**

Prospective cohort study design, with a “within-subject” control procedure.

**Study Sample:**

Twenty adults over-65 post-lingually deafened CI users. All subjects were required to be native Italian speakers, to have normal cognitive level, have no significant psychiatric conditions and/or diagnosed incident dementia, and used CI for at least 9 months.

**Materials and Methods:**

Twenty participants were assessed using the Hearing Handicap Inventory for the Elderly (HHIE), the Geriatric Depression Scale (GDS), and the Speech, Spatial, and Qualities of Hearing Scale (SSQ) before, during, and after ACE program, with a one and 6-month follow up. The cognitive and audiological evaluation was carried out before commencing the ACE program.

**Results:**

The ACE program had a positive impact by reducing HL’s social/emotional effects. Participants benefited from a rehabilitative approach by improving multilevel skills: comprehension of audiological and hearing dimensions, acquisition of communicative, pragmatic and problem-solving strategies, and interaction and sharing of experiences with peers.

**Conclusion:**

Although targeting the older adults with moderate HL, the ACE program also seemed to benefit older adult CI users. An improvement in social and emotional adaptation to hearing difficulties can, in turn, significantly promote optimal use of CI in the older adults, thereby possibly reducing the risk of losing motivation and engagement in device use and in rehabilitation protocols.

## Introduction

Age-related hearing loss (HL) is the third most frequent chronic disease in the older adult population, immediately after cardiovascular disease and arthritis, affecting more than 40% of the population over 65 years of age (Covelli et al., 2015). ARHL determines a complex deterioration processes involving both peripheral and central auditory processing. Consequently, typical ARHL patients experience consistent difficulties in speech conversation and intelligibility in noisy environments and often present aberrant patterns of growth in loudness as sound intensity increases, known as recruitment. This aspect may add to further difficulties in understanding conversations in challenging conditions (Gratton and Vázquez, 2003; Covelli et al., 2015). It is essential to consider its consequences in terms of reduced perception and communication that will inevitably impact on participation in social life, cultural aggregation activities, and relationships. That, in turn, might trigger worsening effects on psychological and/or neuropsychiatric clinical conditions, such as the risk of premature cognitive deterioration, incident dementia and depressive disorders (Lin, 2011; Lin et al., 2011, 2013; Gallacher et al., 2012; Quaranta et al., 2014). There is a body of evidence that declining motivation to communicate may ultimately impede on participation in social life, relational, cultural, and aggregation activities (e.g., Heine and Browning, 2002; Kramer, 2005; Hickson et al., 2006, 2007a,b; Boothroyd, 2007; Lin et al., 2011; Covelli et al., 2015).

Furthermore, when HL occurs later in life, it can possibly coincide with other age-related health problems. Hearing impairment adds to an already compromised functional framework, further negatively affecting sense of independence and autonomy. It can cause everyday restrictions in social participation, feelings of sadness, isolation, helplessness, incompetence, and depression. HL in the older adults may significantly diminish psychological functioning and social interactions (Heine and Browning, 2002), increasing feelings of worthlessness, inadequacy, and isolation, and consequently worsening the quality of life. In the guidelines of mental health services for deaf and hard of hearing people (Trychin, 2002), the author offered a description of the psychosocial consequences of HL classifying five primary dimensions: emotional dimension (shame, depression, anxiety, and frustration); cognitive dimension (lowering of attention and concentration, increased listening effort with more resource-demanding activities, reduction in self-confidence, and **s**elf-efficacy); social dimension (withdrawal, avoidance, and social discomfort), behavioral dimension (limitation/reduction in activities), and physical dimension (headaches, sleep disorders, and sense of strain).

In severe/profound HL cases, when the benefits derived from hearing aids are poor or absent, a cochlear implant (CI) provides the most suitable solution. CI is a sensory neuro-prosthetic device representing the “gold standard” in the treatment of deafness. It works through electrical neuronal stimulation capable of imitating the natural physiology of sensory organs. It offers an auditory threshold in the free field of 20–35 dB for frequencies from 250 to 8,000 Hz and provides an excellent understanding of speech in acoustically quiet environments. When CI is activated, it generates an electrical response, targeting spiral ganglion, and auditory nerve fibers, bypassing the organ of Corti. The brain interprets the artificial electrical stimulation of CI as an auditory sensory input innervating the receptor cells.

Although there is significant variability in CI outcomes in older people—greater than in younger age groups (Schafer et al., 2021), the older adults were shown to benefit from cochlear implantation in terms of restoring auditory functions and enhancing the potential for communication (Capretta and Moberly, 2016). Nevertheless, auditory restoration through hearing aids and/or CI may not be enough to address the psychological, emotional and relational consequences of deafness (Boothroyd, 2007).

Over recent years the adoption of the modern International Classification of Functioning, Disability and Health (ICF) approach (World Health Organization, 2001) has led clinicians to consider a multidisciplinary approach to treating the hearing-impaired older adults. This approach focuses on audiological, communicative, cognitive, and psychological aspects. In this way, the ultimate goal of multimodal interventions is essentially that of concretely improving their quality of life, reducing communicative, social, and emotional limitations imposed by sensory deprivation.

Additionally, research into treating severe-profound deafness in adults and older adults has increasingly highlighted that hearing impairment in old age needs to be tackled beyond aural rehabilitation through hearing aids and/or CI alone (Rosenhall, 2001). The necessity for supplementing rehabilitation with interventions, training, and programs on communicative and psychosocial needs has been increasingly recognized (Kricos et al., 1992; Rosenhall, 2001; Hickson and Worrall, 2003; Boothroyd, 2007).

Active Communication Education (ACE) is a rehabilitation program focusing on the quality of life and psychological status of older people with hearing impairments developed through a research program at the University of Queensland (Hickson and Worrall, 2003; Hickson et al., 2007a).

The original version of ACE was a program of five weekly sessions created to help adults with HL (hearing aid users and non-users) become more effective communicators in order to improve their quality of life. The ACE handbook defines it as a problem-solving interactive program “to help adults with hearing loss to become more effective communicators and to provide them with strategies to cope with everyday difficulties” (Hickson et al., 2015, p. V).

Studies exploring the effectiveness of ACE in older persons with mild-moderate HL with and without hearing aids demonstrated the ACE program to be effective in reducing communication difficulties (Hickson et al., 2007b; Oberg et al., 2014a; Öberg, 2017; Rivera et al., 2020). The successful original Australian version (Hickson et al., 2007b) was modified and translated into Swedish (Oberg et al., 2014b). Recently an adaptation into Spanish for use with Chilean older adults with HL (Rivera et al., 2020) has been published. Authors conducted a pre-post single-blinded exploratory cohort study on a sample of 66 older adults with HL and not wearing hearing devices. At the end of the program, consistent with previous research into ACE effectiveness, participants showed a significant reduction in HL’s social and emotional impacts and an enhancement to hearing functionality for daily-life activities.

Nevertheless, all these studies investigated the effectiveness of ACE at reducing communication difficulties and hearing handicaps in subjects with a mild or moderate hearing impairment, indifferently using or not using hearing aids. None of these studies investigated the effectiveness of an ACE program on profoundly deaf older adults with CI.

The aims of the present study were:

(i) Firstly, to investigate the effects of the ACE program (Hickson et al., 2007a) on older adult CI users’ quality of life in terms of the socio-emotional impact of HL, through Hearing Handicap Inventory for the older adults (HHIE; Ventry and Weinstein, 1982; Ralli et al., 2014).
(ii) The secondary outcomes were to explore, if present, any changes in perceived auditory disability and in depressive symptomatology, and to investigate the influence of audiological and personal variables of the intervention outcome.

## Materials and Methods

### The Active Communication Education Program for Italian Older Adults Cochlear Implant Users

The preliminary steps in the present project were to translate and adapt into Italian the Active Communication Education (ACE) program (Hickson et al., 2007a) designed to improve metacognitive awareness, problem-solving and self-management in adults with hearing impairment.

To translate and adapt ACE into Italian and be cognizant of Italian culture, we followed the guidelines recommended by Hall et al. (2018).

#### Below Is a Procedural Summary

(i) Initially, we researched the literature to establish if an Italian translation of the ACE program already existed: two authors (IG and PM) searched independently and no Italian translation was found.
(ii) Three authors (IG, MN, and PM), who have broad experience with CI users, set out the key objectives to be followed and customized for the end-users. To achieve this, each of the three authors prepared a list of characteristics for the target population (older adult CI users) from cultural, linguistic, and audiological points of view.
(iii) Three of the authors (IG, MN, and PM) independently translated the entire ACE program (handbook and handouts). Italian is the mother tongue for all three. All of them are health care professionals with wide experience in the management and rehabilitation of hard-of-hearing older patients (an audiologist, a speech therapist, and a psychologist). Moreover, one of the authors (PM) has a certified proficiency linguistic competency, and the other two (IG and MN) have excellent proficiency in English.
(iv) The obtained three draft translations were compared, discrepancies were reviewed and harmonized, and one single translation/adaptation was finally created. A further comparison of the translated document with the original was undertaken by an author of the study (BI) who was not involved in the draft translation process. No significant discrepancies were found, and the translation process was completed.
(v) In order to meet the characteristics and needs of the target population (older adult Italian CI users) some linguistic, cultural, and structural modifications were required. Table 1 summarizes the main modifications.

**TABLE 1:** Summary of main modifications compared to the original ACE program.

Type of modification		Motivation
Length of program and frequency of sessions	Two modules were added to the original 5, giving a total of 7. Sessions were fortnightly.	The program was aimed at the older adults with CI: an introductory module was added to give information on the correct management and functioning of CI (module 1). A further module was added after the 4th to give more time for participants to practice assertive skills and communication strategies. The fortnightly frequency was decided to allow participants to have more opportunities to practice the strategies and to meet participant organizational needs.
Content of sessions	Modification of the content of modules 5 (listening to other signals) and 7 (usability of advance CI technology)	The general objectives of the two modules were maintained but adapted to the technology in use (i.e., bimodal mode), with specific information concerning Assistive Listening Devices (MODULE: “usability of advance CI technology”). Clinical specialists for all CI brands worn by the participants were involved in these sessions.
Linguistic modification	Partial modification of handout 26: speechreading (section linguistic factor) and lipreading exercise (spin test)	Only phonetic, syntactic and morphological changes were made to make exercises suitable for Italian-speaking participants. The adaptation was undertaken by the audiologist and speech therapist who participated in the study.

After the translation and the reported changes were completed, the Italian ACE version for CI older adult users was delivered through seven fortnightly group sessions lasting about 2 h each and for up to six-eight participants. A psychologist, a speech-therapist, and an audiologist, all with a significant experience in the management and care of HL, conducted the group sessions.

The aims of each session were the same as the original ACE program (Hickson et al., 2007a): expression of communication needs, awareness of obstacles to communication, problem-solving approach, and metacognitive control empowerment.

The expression of communication needs’ outcome was delivered during the first two modules, which focused on introducing the program and identifying participants’ personal and specific communication needs or difficulties in their daily use of CI. The needs and communication requirements identified during these first two sessions determined the subsequent priorities for addressing these issues. Each session topic was selected, starting with the participants’ communication needs deemed as most important.

Regarding the “awareness of communication obstacles” outcome, there was a detailed discussion on the possible causes of the expressed participant communication difficulties and their possible solutions. Participants’ awareness was developed by performing practical activities during the session or by giving each participant the relevant information to perform these at home.

Participants were encouraged to use the problem-solving and communication strategies practiced during each session in a wide range of communicative challenging situations.

Metacognitive control can be defined as the ability to self-evaluate the accuracy and adequacy of one’s performance during mental or operational tasks. It includes self-instruction skills, i.e., being conscious of when, how, and why to flexibly apply one strategy or another when trying to reach one’s goals. It also includes awareness of the resources available and their limitations, personal strengths, and weaknesses (Kluwe, 1982). To empower the metacognitive control, sessions were structured so that the communication activity (characteristics, difficulties, and solutions) was brought under the conscious control of each participant, through demonstrations, practical exercises, and observations of others’ alternative behavior when faced with similar problems.

Finally, in each session, the presence of family members and/or significant others was highly encouraged and, when present, they were actively engaged in the sessions.

### Participants

Participants were recruited from the Cochlear Implant Center, Department of Sense Organs, La Sapienza University of Rome. The study was approved by the Policlinico Umberto I Ethical Committee (rif. 5982, 22.04.2020).

Inclusion criteria for the study were: age at the time of study being 65 years or over; use of unilateral (CI), bilateral (CI/CI) or bimodal (CI/HA) cochlear implants; duration of CI use > 18 months; absence of malformations of the inner ear, ossification/fibrosis of the cochlea and/or incomplete insertions of the array; no significant self-reported history of psychiatric conditions and/or diagnosed incident dementia; average cognitive level (established as being ≥ 25 percentile at Raven Colored Progressive Matrices-CPM (Raven, 1986); the commitment to participate in at least 60% of the ACE program, as indicated by Hickson et al. (2007a).

In total, 30 CI users aged 65–81 years were identified and 24 of them agreed to participate in the study. Before participating in the study, all subjects were required to complete an informed consent form describing the assessment and treatment procedures.

During the program, four subjects were excluded from the study because they did not attend sufficient sessions to be included in the analysis. More specifically, two subjects have attended only 30% of the sessions because of serious illness of a family member; one gave up after two sessions because of cholesteatoma on the implanted ear, and one due to fracture of the femur. Therefore, the final number of participants who completed the protocol was 20 (9 men and 11 women), with a mean age of 72.05 years (range 65–81 years; *SD* 5.52). All unilaterally and bilaterally implanted participants presented with bilateral severe-profound HL. Bimodal users showed severe/profound HL on the implanted side and a downsloping moderate-severe HL on the HA side.

Fourteen of the subjects were married; four were widows and two were unmarried. Of those unmarried and widows, four lived alone and two lived with siblings. All subjects were implanted by two expert surgeons using a traditional implant, with a receiver housed posteriorly to the mastoid, mastoidectomy and posterior tympanotomy, and cochleostomy at the antero-superior margin of the round window. Five participants (25%) were unilateral CI users, four (20%) bilateral and 11 (55%) bimodal (CI/HA). The mean age at cochlear implantation was 67.7 years (range 59–81; *SD* 6.0) and the mean time of CI use was 5 years (range 1.6–16 years; *SD* 3.92). All participants received 6 months of auditory training soon after CI activation, as is our Cochlear Implant Center practice. The subjects’ Free Field (FF) threshold mean value was 28.3 (*SD* 1.8) dB HL from 250 to 4,000 Hz.

Education level was measured according to the International Standard Classification of Education (ISCED-11) of the 20 participants, seven (35%) had only primary education, five (25%) had an upper secondary education and eight (40%) achieved a bachelor’s or equivalent level. Descriptive data of participants are shown in Table 2.

**TABLE 2:** Descriptive data of the participants (*n* = 20).

Personal variables		Mean (*SD*) [range]
Age at test (years)		72.05 (5.52) [65–81]
Duration of hearing loss (years)		36.00 (16.73) [2–70]
Age at CI (years)		67.7 (6.0) [59–81]
CI use (years)		5 (3.92) [1.6–16]
Level of education (years)		8.65 (3.57) [5–18]
		***n*** **(%)**
CI mode	Unilateral	5 (25)
	Bilateral	4 (20)
	Bimodal (CI/HA)	11 (55)
Sex	Male	9 (45)
	Female	11 (55)
Status	Married	14 (70)
	Unmarried	2 (10)
	Widow	4 (20)
	Living alone	4 (20)
	Living with significant others	16 (80)
Socioeconomic status	Low	2 (10)
	Medium	16 (80)
	Medium-high	2 (10)

**FIGURE 1:**
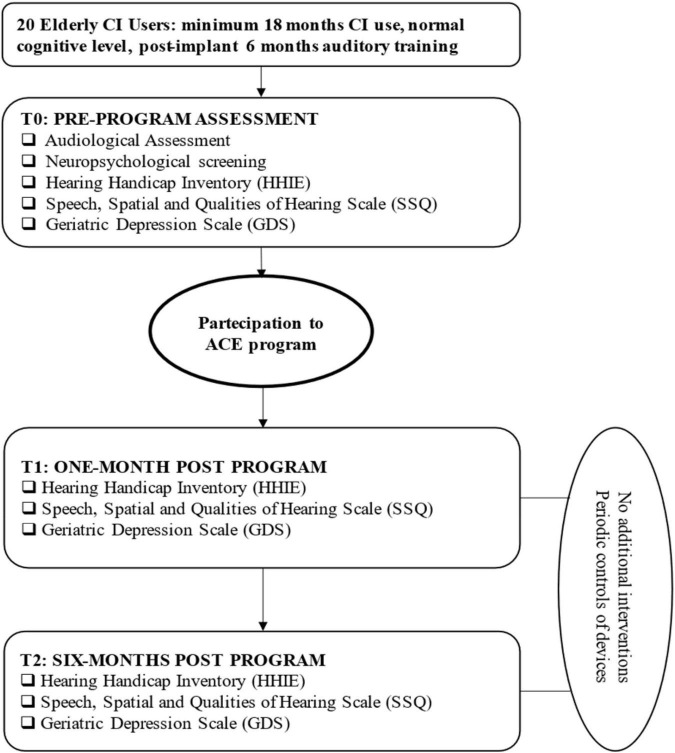
Flowchart of patient assessment during the ACE program.

The evaluation of audiological characteristics was carried out in daily listening mode before commencing the ACE program (see flowchart in Figure 1). Both HA and CI fittings were individually controlled for each recipients before testing. Most comfortable levels of CI and HA were balanced and confirmed to be appropriate when listening bimodally/bilaterally to avoid any discomfort due to loudness summation effects (Mancini et al., 2021). The audiological assessment was performed in a sound-proof audiometric chamber through an Aurical audiometer (Otometrics Taastrup, Denmark) connected to two loudspeakers placed at 0° azimuth at a 1-m distance from the participant’s head. The speech perception tests consisted of phonetically balanced bisyllabic words and sentences (Cutugno et al., 2000) and the Italian Matrix Test (It Matrix; Oldenburg Italian version; Puglisi et al., 2015).

The bisyllabic words and sentences were presented in quiet and in fixed signal/noise ratio (SNR) + 10. The speech material was highly redundant, the primary signal presented at 0° from the participant’s head at 65 dB HL. The results are reported in% correct score but were further converted into Rationalized Arcsine Units (RAU), used to transform data obtained from speech intelligibility tests to make them suitable for parametric statistical analyses (Studebaker et al., 1995). RAU scores are often used in audiology research statistical analysis to avoid the ceiling and floor effects (Studebaker et al., 1995).

The It-Matrix was based on an adaptive SNR paradigm and measured the speech reception threshold (SRT), where 50% of sentences were repeated correctly. Because of the semantically unpredictable structure, the lists cannot be memorized easily and thus, can be used repeatedly. Participants were advised to ask for a break whenever they needed one to avoid performance deterioration due to excessive strain. A training scheme was created for each test and provided to all participants before undertaking the test. The outcomes for free speech perception in quiet and in noise for the study group are reported in Table 3. It is noteworthy to report that all subjects could perform speech perception in quiet, although with variable outcomes. On the other hand, 10% (2 participants) and 15% (3 participants) were not able to complete the test at fixed SNR, and 30% scored > 10 dB SNR at Matrix test. The data concerning neuropsychological status were collected by administering the Repeatable Battery of Assessment of Neuropsychological Status tool (RBANS; Randolph et al., 1998; Ponteri et al., 2007; Randolph, 2007). It is an easy-to-use and relatively quick neuropsychological screening tool (about 40–50 min) consisting of 12 tests designed to evaluate five cognitive domains (immediate memory, visuospatial-visuoconstructive skills, language, attention, and deferred memory). In Table 4, the mean RBANS total score and subscale scores are presented.

**TABLE 3:** Speech perception scores median values for the study group. Speech perception % values were converted into RAU score (rationalized arcsine units) for statistical analysis.

Test		Score% median (min-max)	RAU Score median (min-max)	SRT dB SNR median (min-max)
Words	Quiet	79 (32–100)	77.25 (33.71–112.77)	–
	SNR + 10	52.50 (0.00–85.00)	52.21 (–12.77–83.99)	–
Sentences	Quiet	88.50 (33.00–100.00)	88.42 (34.65–112.77)	–
	SNR + 10	56.50 (0.00–97.00)	55.78 (–12.77–102.11)	–
It-matrix		–	–	8.30 (–0.50–20.00)

*SNR, signal-to-noise ratio. It is the ratio between the intensity of a signal and the intensity of the background noise. A ratio greater than 0 dB or higher than 1:1, signifies more signal than noise. SNR + 10 = The intensity of the signal is 10 dB higher than noise level.*

*RAU, Rationalized Arcsine Units; SRT, speech reception threshold.*

**TABLE 4:** Mean and standard deviation of the RBANS scores in pre-program assessment.

RBANS domain	Mean score (*SD*)	Descriptive results
Immediate memory	95.1 (*SD* 17.2)	Average
Visuospatial ability	95.9 (*SD* 12.2)	Average
Language	88.4 (*SD* 9)	Low average
Attention	86.7 (*SD* 16.2)	Low average
Delayed memory	98.7 (*SD* 13.5)	Average
General score	88.2 (*SD* 12.03)	Low average

### Outcome Measures

Participants’ quality of life, in terms of the social and emotional impact of HL on daily lives, was assessed at baseline, prior to the start of the ACE program (T0), within 1 month of the end of training (T1), and 6 months afterward (T2) (flowchart in Figure 1). It was not possible to have a control group due to the large intersubject variability in CI users and the difficulties associated with selecting a control group of sufficient numbers balanced in terms of factors that could affect their composition (i.e., subject age, duration of deafness, device type, baseline performance). It was decided that a “within-subject” control procedure with each subject serving as his or her own control would be adopted. We followed the approach used by Oba et al. (2013), where attention was given to collecting data from extensive baseline performance measures. Baseline performances were repeatedly assessed once per week for a minimum of four sessions. Oba et al. (2013) indicated that if performances measured at the fourth session improved by more than one standard deviation from the first three sessions, baseline performances were assessed a fifth time. The scores of the fourth and fifth test sessions were then averaged and compared to the pretraining baseline scoring.

Tests used for the participants’ assessments are listed and described below.

#### The Hearing Handicap Inventory for the Elderly

As for the primary outcomes measure, the Socio-Emotional wellbeing outcome was assessed using the Hearing Handicap Inventory for the elderly (HHIE; Ventry and Weinstein, 1982; Ralli et al., 2014). HHIE is a standardized self-reporting questionnaire, designed to assess the effects of hearing impairment on emotional and social adaptation in the older adult population. The aim is to identify self-perception of emotional and situational handicaps caused by hearing impairment (as distinct from objective audiological functioning), considering that the correlation between hearing impairment levels and handicap levels is not always linear (Ventry and Weinstein, 1982). The tool, widely used as a measure of quality of life in older adults with HL, consists of 25 questions, where it is necessary to select one of three available options (“yes,” “sometimes,” “no”) delivering three possible results: level of perceived emotional distress HHIE-E (Emotional Subscale), level of perceived social distress HHIE-S (Social Subscale) and the total level of perceived distress HHIE-T (Total Subscale) as related to their personal hearing impairment condition.

Scores are expressed as percentages by dividing the raw score obtained at each subscale by 100. Interpretation of scores is as follows: 0–16% suggests no self-perception of handicap caused by hearing impairment; 18–42% suggests mild-moderate hearing handicap; > 44% suggests the presence of significant perception of handicap caused by hearing impairment (Newman et al., 1991).

#### Speech, Spatial, and Qualities of Hearing Scale

As for secondary outcome measures, the Speech, Spatial, and Qualities of Hearing Scale (SSQ) (Gatehouse and Noble, 2004) was used.

SSQ is a self-reporting questionnaire designed to measure a variety of hearing disabilities in a range of different contexts and realistic communication scenarios.

SSQ is an intermediate link between the audiological measurement of someone’s HL and a patient’s self-assessment of how HL impacts their wider life (i.e., their handicaps or participation restrictions). Particular attention is given to the competitive, spatial, and movement components of spatial hearing and the real auditory world’s three-dimensional and temporally dynamic aspects. SSQ was developed assuming that hearing is “scenic analysis”: sound always occurs around us virtually, emanating from different sources and at several times. When a sound is salient, the listener shifts attention, with eyes and head toward the source. One listens carefully; thus, one comprehends sound and can engage in communication and effective dialogue. SSQ consists of three sections:

– Section one (Speech): 14 items on speech hearing. Items covered include several speech hearing situations: the condition of competing sounds, visibility of talkers, number of persons included in the conversation, and different background conditions.
– Section two (Spatial): 17 items on spatial hearing: items covered include directional and distance judgments and discrimination of movement.
– Section three (Quality):18 items on other hearing qualities: items included ease of listening, naturalness, clarity, and identifiability of different speakers, signal segregation, identification/recognition of different musical pieces and instruments, and different everyday sounds.

The questionnaire is best administered in the form of an interview (Gatehouse and Noble, 2004), and participants rated their communication performance in each situation with a score of 0–10 (visual analog scale, VAS), with higher scores always reflecting greater ability (or less disability). All subjects were advised that “10” indicated that they could perform the situation perfectly, whereas “0” meant they could not perform the situation. In addition, the option “not applicable” could be checked in cases where the question did not represent an everyday situation.

#### The Geriatric Depression Scale

As for secondary outcome measures, the Geriatric Depression Scale (GDS) (Yesavage et al., 1982; Galeoto et al., 2018) was used. GDS is a self-reporting questionnaire widely used to evaluate depressive symptoms in the older adults and is also used in cases of mild or moderate dementia. GDS consists of 30 standardized items with two alternative questions (yes/no); the tool excludes the detection of somatic and psychotic symptoms. Each answer is assigned a dichotomic 0/1 score with the final score categorized as follows: 0–9 (absence of depressive symptoms); 10–19 (presence of mild depressive symptoms); and scores above 20 (presence of significant depressive symptoms).

### Statistical Analysis

Analyses were conducted using non-parametric statistics: Wilcoxon signed-rank test (pre-program vs. 1-month post-program vs. 6-months post-program). Bonferroni correction was used to account for the within-group comparisons. Within-group changes and effect sizes (Rosenthal r_equivalent) (Rosenthal and Rubin, 2003) were calculated. The relationships between the personal and audiological characteristics of the study samples and the outcome measures were investigated using the Spearman Rank Correlation Coefficient. Analyses were carried out using a PC version of Statistical Package for Social Sciences 15.0 (SPSS, 8 Chicago, IL, United States).

## Results

### Primary Outcome: Hearing-Related Quality of Life

HHIE subscales and total scores were analyzed. Concerning the Emotional Subscale of HHIE (HHIE-E), at T0, 11 subjects (55% of participants) had a significant emotional maladjustment to HL, six subjects (30%) showed a mild-moderate maladjustment, and three subjects (15%) revealed a decent emotional adjustment to HL.

At T1 and in the 6-month follow-up (T2), 11 subjects (55% of participants) showed mild-moderate emotional maladjustment, six subjects (30% of participants) revealed no hearing-related emotional problems and only three subjects (15% of participants) still had severe emotional impairment.

Concerning the Social Subscale of HHIE (HHIE-S), at baseline (T0), 14 subjects (70% of participants) showed significant social maladjustment concerning their hearing impairment, five subjects (25% of participants) revealed a mild-moderate social suffering and only one subject (5%) had no hearing-related social problems.

At the within T1 assessment, the percentage of subjects with severe social issues dropped to 25% (5 participants), 12 subjects (60% of participants) had a mild-moderate social maladjustment, and three subjects (15% of participants) reported no hearing handicap or social suffering.

At T2, the percentage of participants with severe social problems was unchanged from T1 (five subjects, 25%), 10 subjects (50%) had a mild-moderate hearing or social hearing handicap, and five subjects (25%) reported no hearing handicap or social suffering.

At baseline, the observed mean scores for HHIE were: 45.5 (*SD* 23.5) for the Emotional Subscale (corresponding to significant handicap); 52.9 (*SD* 19.6) for the Social Subscale (corresponding to significant handicap) and 49.1 (*SD* 20.3) for Total Score (corresponding to significant handicap). At T1, the study group obtained a mean HHIE score of 26.0 (*SD* 16.2) for the Emotional Subscale (corresponding to mild-moderate handicap); 32.7 (*SD* 14.5) for the Social Subscale (mild-moderate handicap) and 29.6 (*SD* 14.4) for Total Subscale (mild-moderate handicap**).** For each subscale, score differences between T0-T1 were > 12 points.

At T2 mean scores were: for Emotional Subscale 23.4 (*SD* 16.3); for Social Subscale 32.7 (*SD* 17.9), for Total Subscale 27.4 (*SD* 16.19). Differences T1-T2 were < 12 points, denoting stability of outcomes.

HHIE scores were not normally distributed (Shapiro–Wilk test, *p* < 0.05), therefore, non-parametric statistics were used to explore the questionnaire results.

Wilcoxon signed-rank test for within-group comparisons (pre-program vs. 1-month post-program vs. 6-month post-program) showed statistically significant differences. In Emotional Subscale differences were found between measures at T0 and T1 (*Z* = 3.33, *p* < 0.001) and in T0-T2 comparison (*Z* = 3.35, *p* < 0.001). No statistical differences were found in T1-T2 evaluations (*Z* = 1.72, *p* = 0.084).

In Social Subscale, significant differences were found between T0 and T1 (*Z* = 3.62, *p* < 0.001) and between T0 and T2 (*Z* = 3.52, *p* < 0.001); as for the Emotional Scale, no statistically significant differences were found in T1-T2 comparison (*Z* = 0.65, *p* = 0.51).

In Total Scale significant differences were found in T0-T1 comparison (*Z* = 3.52, *p* < 0.001), in T0-T2 comparison (*Z* = 3.65, *p* < 0.001) and slightly in T1-T2 (*Z* = 1.97, *p* = 0.048).

The effect size for the comparison T0-T1 is medium-large, for Emotional (0.74), for Social (0.81) and for Total Subscale (0.79) (Tables 5A,B).

**TABLE 5A:** HHIE scores and *p*-value with Bonferroni corrections.

HHIE subscales	T0 (*n* 20) % (*SD*)	T1 (*n* 20) % (*SD*)	p	Δ T0–T1	T2 (*n* 20) % (*SD*)	*p*	Δ T1–T2
Emotional	45.5 (23.5)	26 (16.2)	0.003	19.5	23.4 (16.38)	0.08	2.6
Social	53 (19.6)	32.7 (14.5)	≤0.001	19.3	32.7 (17.9)	0.51	0
Total	49.1 (20.3)	29.60 (14.4)	≤0.001	19.5	27.45 (16.19)	0.14	2.15

*Scores are expressed as a percentage by dividing the raw score obtained at each subscale by 100. Interpretation of scores is as follows: 0–16% suggest no self-perception of handicap caused by hearing impairment; 18–42% suggest mild-moderate hearing handicap; > 44% suggest presence of significant perception of handicap caused by hearing impairment. Pre-intervention (T0), 1-month (T1) and at 6-months (T2) follow-up.*

**TABLE 5B:** Effect size for HHIE comparisons at pre-intervention (T0), 1-month (T1) and at 6-month (T2) follow-up.

Variable	Scale	Wilcoxon signed rank test	*T*	*Z*	*p*	Effect size
HHIE	Emotional	T0_T1	9	3.33	**≤0.001**	0.74
		T1_T2	40	1.73	0.08	0.38
		T0_T2	15	3.35	**≤0.001**	0.75
	Social	T0_T1	5	3.62	**≤0.001**	0.81
		T1_T2	70.5	0.65	0.51	0.15
		T0_T2	10.5	3.52	**≤0.001**	0.79
	Total	T0_T1	10.5	3.52	**≤0.001**	0.79
		T1_T2	46	1.97	**≤0.05**	0.44
		T0_T2	7	3.65	**≤0.001**	0.82

*P-values statistically significant.*

### Secondary Outcomes

**FIGURE 2:**
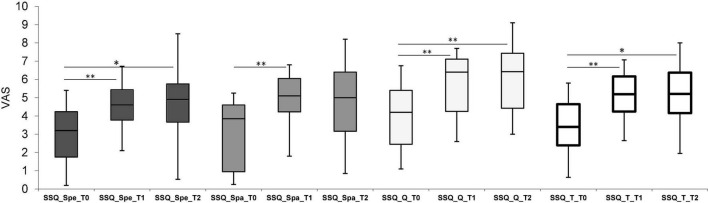
Box plot representing scores obtained for the Speech, Spatial, and Quality (SSQ) questionnaire. Spe, Speech; Spa, Spatial; Q, Quality sections. White bars represent the total scores (SSQ_T), calculated as the sum of all sections. Scores were reported for each follow-up which were, respectively: Pre-Intervention (T0), 1-month (T1) and 6-month (T2). *p ≤ 0.005, ^**^p ≤ 0.001 with Bonferroni corrections.

Significant differences were also found in the SSQ questionnaire (Figure 2). Statistical comparisons between follow-ups showed a global improvement (SSQ_Total) in all three dimensions of the auditory scene 1 month after treatment (T0-T1). Specifically, the results of the Wilcoxon tests indicate: Speech (SSQ_Spe) *p* < 0.001, effect size = 0.87; Spatial (SSQ_Spa) *p* < 0.001, effect size = 0.88; Qualities (SSQ_Q) *p* < 0.001, effect size = 0.88. The scores even improve at T2 for the Speech scale (Wilcoxon signed-rank test *p* = 0.006, effect size = 0.68) and the Qualities scale (Wilcoxon signed-rank test *p* < 0.001, effect size = 0.82) (Figure 2). SSQ_T for unilateral, bimodal, and bilateral users were, respectively: 3.2 (0.7–3.9), 2.8 (0.6–5.8) and 4.6 (3.1–4.7) at T0; 4.5 (3.7–5.2), 5.6 (2.6–7.1), and 6 (5.2–6.2) at T1; 4.5 (3.8–4.8), 5.6 (2.6–7.3), and 6 (1.9–8) at T2. The differences in the results of the SSQ subscales between the three listening modes (unilateral, bimodal, and bilateral) at any time-point were not statistically significant.

Concerning depressive symptomatology assessed using GDS, at baseline, nine subjects (45% of participants) had mild-moderate depressive symptoms, six subjects (30% of participants) had no depressive symptoms, and five subjects (25% of participants) presented significant depressive symptoms. At T1, 15 participants (75%) showed no presence of depressive symptoms, four (20%) had mild-moderate symptomatology and one participant still had significant depressive symptoms (5%). At T2, 13 participants (65%) showed no depressive symptoms, and seven (35%) had a mild-moderate depressive symptomatology. None of the participants showed any significant depressive symptoms. Wilcoxon signed-rank test for within-group comparisons showed statistically significant differences between measures at T0 and T1 (*Z* = 2.93, *p* = 0.003) and between T0 and T2 (*Z* = 2.80, *p* = 0.005) with a medium effect size (T0-T1: 0.65; T0-T2: 0.62).

The final aim of the study was to define the weight of baseline personal and audiological variables on the primary HHIE outcome at T0, T1, and T2.

Regarding personal variables (age, gender, educational level, and GDS), no significant relationships were found at any time point (all *p*-values > 0.1), except for a significant relationship between GDS and HHIE-Emotional and Total Subscale at T0 (*p* < 0.05).

As to audiological variables (duration of HL, of CI use, speech perception outcomes for words and sentences in quiet and SNR + 10 and Matrix tests measured at T0), the correlation coefficients are reported in Table 6.

**TABLE 6:** The correlation between Hearing Handicap Inventory for older adults (HHIE) and personal and audiological variables of the group (*N* = 20).

	HHIE-E T0	HHIE-E T1	HHIE-E T2	HHIE-S T0	HHIE-S T1	HHIE-S T2	HHIE-T T0	HHIE-T T1	HHIE-T T2
Age	–0.16	0.25	0.19	0.15	0.35	0.31	–0.08	0.32	0.25
Education	0.21	0.09	–0.01	0.18	–0.06	0.13	0.18	0.03	0.05
GDS	0.55*	0.41	0.21	0.44	0.17	0.35	0.55*	0.31	0.25
Duration of HL	0.13	–0.04	–0.17	0.24	0.07	0.12	0.23	–0.01	–0.08
CI use	–0.08	–0.03	–0.24	0.04	0.13	0.08	0.01	0.07	–0.04
Words quiet	–0.41	–0.50*	–0.37	–0.40	–0.18	–0.25	–0.46	–0.41	–0.28
Words SNR + 10	–0.17	–0.005	0.06	0.06	0.19	0.07	–0.05	0.05	0.06
Sentences quiet	–0.35	–0.45	–0.10	–0.25	–0.19	–0.15	–0.36	–0.35	–0.11
Sentences SNR + 10	–0.36	–0.44	–0.44	–0.31	–0.39	–0.43	–0.38	–0.43	–0.43
It-matrix dB SNR	0.27	0.43	0.22	0.23	0.13	0.18	0.36	0.32	0.21

Pre-intervention (T0), 1-month (T1) and at 6-month (T2) follow-up. The values of Spearman’s Rank Correlation Coefficient (r) are marked * for p < 0.05. GDS = Geriatric Depression Scale; SNR = signal-to-noise ratio.

## Discussion

The main aim of the present study was to explore the effectiveness of the ACE program (Hickson et al., 2007a) when considering the emotional and social impacts of HL on the quality of life of older adult CI users, using the HHIE as the primary outcome measure.

ACE is a rehabilitation program developed to improve the communication and quality of life of older people with hearing impairments. The empirical evidence to support the effectiveness of the ACE program on older community-based adults with mild-moderate hearing impairment was first studied by Hickson et al. (2007b). They developed a double-blinded, randomized, controlled study, and found significant improvements in the ACE group’s communication participation, wellbeing, and quality of life. Participants in the program also showed a significant reduction in activity limitations. These positive findings reported by Hickson et al. (2007b) were further confirmed in studies conducted by Oberg et al. (2014a) and Öberg (2017), who enrolled groups of adults with a wide range of ages at intervention (39–82 and 41–94 years, respectively) and various degrees of HL (from mild to severe and from mild to profound, respectively). A similar design was used by Rivera et al. (2020) who recruited a group of older adults (age > 65 years) with unspecified degrees of HL and who did not wear hearing aids.

Consistent with the above-cited studies, results from the present investigation indicate how the ACE program can also be used with severe/profoundly deaf older adult CI users, improving their level of hearing-related socio-emotional adaptation. HHIE subscales showed that the majority of them had at baseline severe emotional and social maladjustments (respectively, 55 and 70% of the sample), and that after the end of the program, that number had dropped significantly (reducing respectively, to 15 and 25%). These changes were reflected in a significant reduction in the mean scores that went from 45.5 (*SD* 23.5) to 26.0 (*SD* 16.2) for the Emotional subscale and from 52.9 (*SD* 19.6) to 32.7 (*SD* 14.5) for the Social Subscale. In the T0-T1 comparison a reduction superior to 12 points was recorded for each subscale –that is the minimum score differential indicated by Newman et al. (1991) as significant.

All the participants enrolled in the present investigation had attained the condition of best CI fitting, had received auditory training and, as indicated by the use of procedures developed by Oba et al. (2013) to collect baseline measures, had stable post CI outcomes before starting the study. Notwithstanding this background, taking part in the ACE groups helped many of them further to improve their perceived benefits from CIs. Although CI is an effective device in the treatment of severe/profound HL (Naples and Ruckenstein, 2020), users are still faced with significant communication difficulties in challenging situations such as noisy environments, talking at distance, or multitasking situations. Moreover, older adult CI users show variable outcomes that are generally poorer than younger adults (Aimoni et al., 2016).

Therefore, it could be speculated from these results that helping all CI users to effectively manage challenging situations, even when they have reached optimal hearing ability, could be considered a key to improvement in patient satisfaction.

Findings from the present study shown remarkable HHIE changes in the Emotional and Social Subscales, denoting respectively, a medium and a large effect size, supporting the positive impact of the treatment (Sullivan and Feinn, 2012). These improvements were greater when compared to the studies of Hickson et al. (2007b) and Rivera et al. (2020), which found only minor treatment effects in “within group” comparisons. These differences in outcomes might partly result from differences in group composition between studies. Hickson et al. (2007b) involved only the older adults with mild-moderate HL, half of whom didn’t wear any hearing technology. Rivera et al. (2020) did not specify the degree of HL of the involved subjects, but as none of them were HA users, it is conceivable that their deafness was neither severe nor profound. Hickson et al. (2007b) discussed that the degree of awareness of hearing difficulties in subjects with mild-moderate HL varies and influences the effectiveness of ACE programs on the perceived emotional distress, social withdrawal, and general restrictions on social participation. Accordingly, they found a significant correlation between the personal attitude to hearing impairment of the participant, as measured pre-training through the Hearing Attitudes to Rehabilitation Questionnaire, and the scores for the Hearing Handicap Questionnaire at the end of the ACE program: greater benefit was perceived by subjects with greater awareness of their hearing difficulties (Hickson et al., 2007b). In the present study, all participants were severally or profoundly deaf, and used a unilateral, bilateral or bimodal CI. Thus, it could be presumed that their perceived hearing difficulties were more serious when compared to subjects involved in Hickson et al. (2007b) and Rivera et al. (2020) studies. Therefore they perceived a greater benefit from ACE.

The present study is the first attempt to use the ACE program in a sample made up exclusively of older adults CI users and therefore was a cohort of profound, severe hearing-impaired older adults with long device user experience and duration of HL. In a descriptive review of the psychosocial effects of group audiologic rehabilitation, Preminger (2007) described how most previous studies demonstrated significant benefits in new hearing aid users only. For example, Brewer (2001) reported a significant effect on group rehabilitation in only 15.8% of long-term HA users. Preminger (2007) and Oberg et al. (2014b) speculated that subjects with long experience of severe/profound HL might progressively develop personal knowledge of their deafness and therefore create possible strategies to cope with it. Some of these strategies can be functional, while others manifest as dysfunctional, without personal awareness of their ineffectiveness. When these dysfunctional strategies are continuously used in everyday interactions, they may become too rigid and crystallized, resistant to change, and show a reduced positive re-adaptation after participating in a rehabilitative program (Preminger, 2007).

In contrast to the studies mentioned above, the positive findings observed in the present study group highlighted how significant changes in both social and emotional domains can still be induced in subjects with long-lasting HL. Programs such as ACE that focus on metacognition and problem-solving skills empowerment, with particular attention given to acceptance, understanding, and active patient cooperation, are used to find and develop personal strategies required to manage hearing devices. ACE sessions are implemented to increase participants’ active engagement in the sessions progressively, thereby enhancing their general knowledge about HL effects and incrementally increasing their awareness of personal communicative strengths or limitations, together with their confidence in metacognitive control of challenging experiences. Non-directive interventions- such as the ACE program- seem helpful in reducing general levels of defensiveness and increasing participants’ cooperation in finding personal strategies to face their hearing and communication difficulties in everyday life. Nevertheless, as Preminger (2007) observed, these differences might be explained by the type of intervention and, when small study groups are involved, by demographic differences of class participants.

A further interesting outcome from the present study was that improvements in social and emotional domains measured through HHIE were maintained at the 6-month follow-up without statistical differences compared to pre-intervention measurements. This result seems consistent with Hickson et al. (2007b), who similarly reported stability of outcome measures at the 6- month follow-up for ACE participants. Similarly, Oberg et al. (2014b) and Öberg (2017) found significant long-term effects for HHIE with no statistically significant changes in the 3-week vs. 6-month evaluations.

Some features of the ACE program may have facilitated the maintenance of the benefits at the level of emotional and social adjustment to hearing difficulties. Among these are paper materials with folders for all participants to take home, the delivery of dossiers at the end of each session with practical exercises to try in everyday social interaction, and summary diagrams on the key concepts. It may be that the opportunity to use the strategies learned during the sessions and rely on written material—personal and personalized—has played a role in supporting the maintenance of results at 6 months.

Among the variables not related to the ACE program, and not described in other studies, it should be noted that all participants, once the training had finished, continued to attend the CI Center for routine checks (audiological visits, CI adaptations, periodic cognitive, and psychological assessments). Constant contact with professionals may have helped maintain their motivation to continue implementing the strategies learned and allow for only a minor decay in the memory trace. However, at the end of the program, the participants were not reminded to use the strategies or to do the exercises learned in the program; the study subjects received equal treatment as the others referred to the CI Center, following a multidisciplinary approach for all patients.

The secondary outcomes of the present study were to explore any change in depressive scores and in perceived auditory disability both at the end of the ACE program and at the 6 month follow-ups and investigate the influence of audiological and personal variables on the outcome of the intervention.

Concerning depressive status, GDS scores revealed a significant reduction in depressive scores from T0 to T1 and T2: it is noteworthy that the percentage of participants with mild-moderate and significant depressive symptoms, taken together, was at 80% at baseline, dropping to 20% in the 6-month follow up. These results seem consistent with Hickson et al. (2007b) and with Oberg et al. (2014b). Both found significant ACE effects on the depressive mood of participants, although they used different measures for detecting the presence of depressive symptoms.

It can also be assumed that, for ACE participants, the opportunity to take advantage of an environment where they could express, compare and share their personal experiences and feelings may have been helpful to them, with the effect of both decreasing distress related to hearing impairment and on depressive symptomatology (Öberg, 2017). Particularly important seems to be the “group dimension” of the ACE program, as it can attenuate those effects that Hétu (1996) defines as the psychosocial consequences of hearing impairment. Hearing-impaired persons might perceive their disability as a “stigma,” i.e., an attribution of prejudice associated with feelings of shame and social inadequacy. Consequently, self-isolation and avoidance of social interactions often become maladaptive coping strategies with which to rely on, contributing to increased depressive symptoms in hearing-impaired people. Taking part in a small group with others suffering from HL and who were of similar age can make it possible for the older adults to experience a condition of belonging, support, and acceptance and, therefore, a decreasing sense of being “stigmatized.” Group activities such as lectures, discussions on unpleasant emotions connected to deafness, role play, and concrete examples can progressively help them to gain a more positive attitude toward others and finally toward themselves (Hétu, 1996).

With respect to perceived auditory disabilities, the participants’ scores in the SSQ questionnaire increased significantly during the post-training assessment, showing an improved self-perception of their auditory function in everyday life listening experiences. Overall, the improvement in SSQ-Total score observed in the present study group was clinically relevant, as shown in a prospective clinical study conducted on older adults subjects with CI (Wick et al., 2020). Soon after the end of the ACE program, participants obtained higher scores in all three dimensions of the auditory scenes analyzed by SSQ, indicating better auditory functioning under challenging scenarios (e.g., presence of competitive messages, multi-talker situations, limited possibility to view talkers’ faces for speechreading), in spatial hearing and in self-perceived quality of sounds. Speech and Qualities scores continued to improve at the 6-month follow up. These findings confirm the results of Rivera et al. (2020), who verified that the ACE program could indirectly affect perceived hearing functioning using the Amsterdam Inventory for Auditory Disability and Handicap. They similarly found a significant improvement in the questionnaire scores following the training, concluding that the ACE program can induce positive changes in listening performance amongst older adults with HL, even if they did not wear hearing technology (Rivera et al., 2020). The specific training that the older adults experienced during the program reinforced their ability to employ successful communication strategies (Hickson et al., 2007a,b). That, together with the opportunity to share and discuss them, can help older hearing-impaired adults to gain confidence in managing the consequences of difficult listening and feel increasingly self-sufficient and confident in using communicative strategies with success (Wallhagen, 2010; Rivera et al., 2020). Finally, the self-perception of success can motivate them to face up to experiences previously avoided or restricted, discovering that they may be able to find strategies to understand verbal messages and communicate effectively. Incrementing the number of social interactions and situations where listening is used effectively can create a virtuous circle and consequently influence the self-perceived listening ability observed in the SSQ (Pedley et al., 2005).

A significant improvement, backed up by a medium effect size, was found in depressive symptomatology between pre-treatment and post-treatment GDS scores, which were maintained at 6-month follow-up. Also, a correlation was found between the depressive symptomatology at baseline and the emotional domain of hearing-related quality of life. This correlation can be explained by published research on this topic (Laudisio et al., 2018) that found an association between depressive symptoms assessed with GDS and reduced QoL; moreover, GDS scores above 9/30 best predict poorly perceived health-related quality of life (Laudisio et al., 2018). It also seems relevant to consider the significant number of studies revealing an association between HL and depressive symptoms in the older adult population. The additional emotional and social burdens experienced by this specific population are linked both to practical and emotional factors related to HL. These factors may predispose them to or increase the risk of depressive outcomes or negative impact emotional vitality (Mener et al., 2013; Contrera et al., 2016).

Other variables that could have influenced outcomes measured in the present study were chronological age, gender, level of education, duration of HL, duration of CI use, and speech perception abilities in quiet and noise.

No significant differences in social/emotional impacts of HL on HHIE outcomes were identified concerning age and gender neither at baseline nor in post-program evaluations. With regard to the effect of age, the present findings appear consistent with the work of Hickson et al. (2007b) and with other studies assessing the effects of single or group audiological rehabilitation (Preminger, 2007). However, the present study contradicts that of Öberg (2017), where significant interaction effects of age and gender were identified regarding HHIE-T and HHIE-S in the short and long-term evaluations. Notwithstanding this, although adopting the same rehabilitative program and the same outcome measures, results are not fully comparable because of significant demographic and audiological differences in the samples. In particular, Öberg (2017) includes a larger sample (*N* = 77) with a complete range of HL (from mild to profound) with an age range from 41 to 94 years, of which 14% were aged < 65 years and 86% aged > 65 years. Moreover, as highlighted by Öberg (2017), it is possible that the statistically significant improvement amongst older—rather than younger participants—may be related to the low statistical power for younger individuals, as they only represented 14% of the sample.

The only factor showing a significant correlation with the Emotional subscale of HHIE, measured soon after the end of the ACE program, was the baseline words recognition score in quiet.

The lack of correlation is likely to be multifactorial, as many factors can influence both hearing performance and quality of life. Effectively, self-assessed hearing handicaps cannot be reduced solely to speech perception-related HL, as HHIE measures typically reflect the self-assessment of handicap across many prior daily life experiences rather than the self-assessment of handicap during or immediately after a speech recognition task.

A further explanation for the lack of correlation is the variability of speech perception ability, ranging from very poor to average for a CI user, and, given the small study group, this may have conditioned statistical analysis. All subjects involved in the study were able to complete the speech perception test in quiet, with scores ranging from 0 to 100%, while up to 30% of subjects could not complete the test in noise, which was particularly evident for the Matrix sentence test. In this respect, the Matrix test is semantically more complex when compared to everyday semantically predictable sentences (Mancini et al., 2021), which might have influenced the scores and the percentage of patients able to complete the test. Furthermore, in the present study, the duration of HL was, on average, 36 years, and the effect of duration of deafness has been shown to be strongly inversely correlated to hearing outcomes, especially when subjects were tested in noise (Mosnier et al., 2014).

Furthermore, outcomes in auditory perception have been shown to be poorer in the older adults when compared to younger individuals, as HL and aging reduce central and peripheral binaural processing, which partially accounts for the difficulties that such subjects experience in complex listening situations (Moore, 2016).

Similar to our findings, an absence of or modest association between auditory perception tasks and HHIE subscales was also reported for pure-tone thresholds (Gates et al., 2003), word recognition in quiet (Weinstein and Ventry, 1983), and in background noise (Gates et al., 2008). Conversely, in a study involving 162 middle-aged to older adults with varying degrees of HL, an association between HHIE and speech recognition specific to sentences with limited semantic context was found (Eckert et al., 2017). These findings are consistent with the observation that the older adults can benefit from contextual semantic cues to a similar extent as that of younger adults (Sheldon et al., 2008).

Differences in findings with the above study should also be read in the light of the specific composition of our study group which was composed of profoundly deaf cochlear implantees. Therefore, it was characterized by additional listening difficulties related to a device that is more limited in frequency resolution and loudness growth when compared to natural hearing (Dorman and Gifford, 2017). Effectively, Park et al. (2011) found similar outcomes concerning the absence of statistically significant correlations between pre-and -post-implant speech recognition in fixed SNR scores and pre-post implant HHIE scores.

To our knowledge, no other research has been published concerning the correlation between HHIE and speech perception tests using adaptive noise paradigms in CI users.

In conclusion, considering the challenging experiences that older adult CI users encounter in everyday communication, the outcomes from the present study and the literature underline the need for rehabilitative programs approaching both audiological and extra-audiological variables. These should include multilevel skills such as comprehension of audiological and hearing dimensions, acquisition of communicative, pragmatic, and problem-solving strategies, implementation of interaction, and sharing experiences with peers. As the number of subjects attending the program increases, this will most likely help in shedding more light on further variables influencing self-perceived disability and further improve the implementation of rehabilitation protocols aimed at promoting optimal use of CI, with high levels of motivation and perceived benefits in the older adult population with HL.

## Limitations

The main limitation of this study was the absence of a control group. The decision not to include a control group was related to statistical/demographical issues concerning the large intrasubject variabilities found in CI users (Oba et al., 2013) which did not allow us to find a good match control. As a consequence, a within-subject control procedure was chosen (Oba et al., 2013). This procedure has rigorous criteria that need to be employed to obtain reliable baseline measures. Nevertheless, it is not possible to state whether the recorded changes at HHIE were effectively induced by program participation rather than spontaneous personal modifications associated with the passing of time (Shadish et al., 2002). Additionally, it is not possible to ascertain whether other kinds of interventions, such as social or cognitive ones, could be equally effective in improving socio-emotional wellbeing in older adult CI subjects (Hickson et al., 2007a; Rivera et al., 2020).

Another potential limitation concerns the tool selected to measure the primary outcome of the present study. HHIE was selected because it is one of the most commonly used questionnaires for assessing hearing-related perceived handicaps and the psychosocial effects of audiological rehabilitation programs (Preminger, 2007). Consequently, it has been used in several studies on the effectiveness of audiological rehabilitation (Hawkins, 2005). In the present study, HHIE was used as the primary measure to assess the level of perceived hearing-related quality of life outcomes in a sample of older adults CI users. Nevertheless, the HHIE questionnaire cannot describe all of the psychosocial consequences of HL or the actual quality of life in absolute terms. The concept of quality of life is broader than just health-related problems. We wholeheartedly agree with Boothroyd (2007) when he states: “Quality of life reflects self-assessment of the current life experience and includes such things as enjoyment, meaning, purpose, usefulness, value, freedom of choice, and independence. Quality of life is a moving target. It is influenced by function, activity, and participation, but is by no means completely determined by them” (p. 64). Preminger (2007) highlighted that HHIE only focuses on the emotional and situational reactions to hearing impairment, but it does not assess the cognitive, interpersonal, and physical reactions related to HL. Finally, although the present study group was homogeneous in composition, the number of older adults CI users was small. Hawkins (2005), in his systematic revision, highlighted how often the number of subjects used in the efficacy studies of adult rehabilitation programs should be characterized as relatively small. This aspect limits the generalization of the present results, and there is a need for other studies with a more significant number of subjects to confirm the results.

## Conclusion

The present study represents the first attempt to evaluate the benefits of the ACE program for older adults CI users. Results are promising as they show a significant decrease in self-perception of emotional and situational distress, an improvement in depressive symptomatology and a reduction in self-perceived auditory disability. The increased awareness of the predictability of some challenging social situations can result in decreased anxiety, insecurity, and social tension (Andersson et al., 1997) and better use of personal resources (Cornoldi, 2011). In this context, this rehabilitative approach can promote the acquisition of communicative and pragmatic strategies to confront daily conversation obstacles. Further, it can increase self-confidence, self- responsibility, and assertiveness. These improvements can significantly support better use of CI in the older adults hearing-impaired population, reducing the risk of losing motivation and engagement in its use and the associated rehabilitation protocols.

Further studies, designed to overcome the limitations of the present study and incremental to the degree of evidence in the present findings would be desirable in future research.

## Data Availability Statement

The raw data supporting the conclusions of this article will be made available by the authors, without undue reservation.

## Ethics Statement

The studies involving human participants were reviewed and approved by Policlinico Umberto I Prot. 259/2020. The patients/participants provided their written informed consent to participate in this study.

## Author Contributions

All authors contributed to the conception, design of the study, data collection, interpretation of results, and approval of the final version.

## Conflict of Interest

The authors declare that the research was conducted in the absence of any commercial or financial relationships that could be construed as a potential conflict of interest.

## Publisher’s Note

All claims expressed in this article are solely those of the authors and do not necessarily represent those of their affiliated organizations, or those of the publisher, the editors and the reviewers. Any product that may be evaluated in this article, or claim that may be made by its manufacturer, is not guaranteed or endorsed by the publisher.
